# Computational Identification of MicroRNAs and Their Transcript Target(s) in Field Mustard (*Brassica rapa * L.)

**DOI:** 10.15171/ijb.1390

**Published:** 2017-03

**Authors:** Behzad Hajieghrari, Naser Farrokhi, Bahram Goliaei, Kaveh Kavousi

**Affiliations:** ^1^ Department of Bioinformatics, Institute of Biochemistry and Biophysics (IBB), University of Tehran, Tehran, 13145-1365, Iran; ^2^ Department of Agricultural Biotechnology, College of Agriculture, Jahrom University, PO BOX 74135-111, Jahrom, 74135-11, Iran; ^3^Department of Biotechnology Engineering, Faculty of Energy Engineering and New Technologies, Shahid Beheshti University G.C., Evin, Tehran,19839-4716, Iran; ^4^Departments of Biophysics and Bioinformatics laboratories, Institute of Biochemistry and Biophysics (IBB), University of Tehran, Tehran,13145-1365, Iran; ^5^Laboratory of Complex Biological Systems and Bioinformatics (CBB), Institute of Biochemistry and Biophysics (IBB), University of Tehran, Tehran, 13145-1365, Iran

**Keywords:** *Brassica rapa*, Expressed sequence tag, Homology search, Non-protein coding RNA, MicroRNA

## Abstract

**Background:**

Micro RNAs (miRNAs) are a pivotal part of non-protein-coding endogenous small RNA molecules that regulate the genes involved in plant growth and development, and respond to biotic and abiotic environmental stresses posttranscriptionally.

**Objective:**

In the present study, we report the results of a systemic search for identification of new miRNAs in *B. rapa* using homology-based ESTs (Expressed Sequence Tags) analysis and considering a series of fi ltration criteria.

**Materials and Methods:**

Plant mature miRNA sequences were searched in non-protein coding ESTs registered in NCBI EST database. Zuker RNA folding algorithm was used to generate the secondary structures of the ESTs. Potential sequences were candidate as miRNA genes and characterized evolutionarily only and if only they fi t some described criteria. Also, the web tool psRNATarget was applied to predict candidate *B. rapa* miRNA targets.

**Results:**

In this study, 10 novel miRNAs from *B. rapa* belonging to 6 miRNA families were identified using EST-based homology analysis by considering a series of fi ltration criteria. All potent miRNAs appropriate fold back structure. Several potential targets with known/unknown functions for these novel miRNAs were identified. The target genes mainly encode transcription factors, enzymes, DNA binding proteins, disease resistance proteins, carrier proteins and other biological processes.

**Conclusions:**

MicroRNA having diverse functions in plant species growth, development and evolution by posttranscriptionally regulating the levels of specific transcriptome so by effecting on their translation products. Research in miRNA led to the identification of many miRNAs and their regulating genes from diverse plant species.

## 1. Background

Varieties of factors in eukaryotes regulate the gene expression during transcription and afterwards. Micro RNAs (miRNAs), non-protein coding RNA molecules consisting of ~22 nucleotides, are a class of such regulators that negatively regulate gene expression. These highly conserved molecules, with no more than 4 mismatch nucleotide substitutions in plants, regulate their target mRNAs within cytoplasm post transcriptionally either by mRNA cleavage or translation inhibition (1,2). Studies have shown that miRNAs regulate large number of genes involved in plant growth and development, environmental stress response, signal transduction as well as pathogen invasion (3-7). RNA polymerase II is responsible for the transcription of MicroRNAs from intergenic and/or intragenic locus/loci, similar to what is seen for protein coding genes. These double stranded primary transcripts known as pri-miRNAs do have a cap and poly A tail (8,9), adopting a hairpin-like secondary structure(s). In plants, miRNA precursors (pre-miRNAs) vary in their sequence length, ranging from 50- 350< nt, appearing in stem-loop structures. These pre-miRNAs are degraded in nucleus by miRNA processing machinery that its core component is RNase III enzyme Dice like 1(DCL1) (10). The strands of the duplex will denature to produce mature miRNA from one strand and miRNA* from the other, each being ~22 nt in length (11). Thus, both strands on either 5^’^ or 3^’^ of the precursor sequence have the potential to turn into mature miRNA (11). The miRNA/miRNA* duplexes are different by 2 nt owing to the staggered cuts of DCL1 at their 3^’^ ends (12). After exporting methylated miRNA/miRNA* to cytoplasm via HASTY (13,14), the miRNA strand of miRNA/miRNA* duplex is preferentially loaded into the ARGONAUTE1 (Ago1) associated RNA-induced silencing complex (RISC) (15). The complex is able to recognize completely or nearly complement sequences in target transcript(s) and initiate cleavage or translational arrest (16). The miRNA*s are then subjected to degradation. Unlike animals with rather weak complementarity between miRNA-mRNA, plant miRNAs demonstrate near to perfect binding to their target mRNA (17).

Identification of miRNAs via laboratory techniques due to the abundance of diverse small non-coding RNAs (ncRNA), siRNA, miRNA and ta-siRNA, are rather difficult and expensive (18). Furthermore, such experimental attempts more often miss quite number of miRNA due to their low abundance. In contrast, computational approaches allow identification and structure prediction of miRNAs. Nevertheless, following such predictions a thorough experimental analysis needs to be set to prove the functionality of such sequences.

Due to high level of conservation in plant mature miRNAs (19), homology based searches seem to be feasible allowing prediction of potential miRNAs and their targets. However, comparative genome-based homology can only identify conserved miRNAs leaving some less-conserved potential sequences with possibly important biological roles behind (20). Thus, in any case both in silico analysis and in vitro techniques need to take into consideration to recover miRNA sequences as much as possible (21-26). Here, field mustard (*Brassica rapa* L.) with currently known 96 miRNAs as released into miRBase (release 21; June 2014) was checked to determine its putative miRNAs via in silico analysis. Such data may allow designing targeted experiments to unravel the biological functions of miRNAs in gene regulation.

## 2. Objectives

Here and according to high sequence conservation among plant species for miRNA families, some conserved sequence and structural features, as well as thermodynamically properties, used as major principals for identification of new miRNAs in *B. rapa* via delving into ESTs (Expressed Sequence Tags) similar to some earlier reports.

## 3. Materials and Methods

### 3.1. Mature miRNA Query Sequences and *B. rapa* ESTs

A total of 7057 sequence of plant mature miRNA from 73 plant species were downloaded from miRNA Registry Database, miRBase (http://www.mirbase.org) current version (release 21; June 2014; (27-29). The reduplicative sequences of miRNA within the plant species were removed to avoid redundant or overlapping miRNAs and only the unique ones were used as query. *B. rapa* mRNA, EST sequences were downloaded from the EST database (dbEST) available in the National Center for Biotechnology Information (NCBI) GeneBank nucleotide databases (http://www.ncbi.nlm.nih.gov).

### 3.2. Availability of Software

BLASTN and BLASTX programs (available at http://www.ncbi.nlm.nih.gov/blast) were used to search *B. rapa* miRNA homologs. For predicting pre-miRNA secondary structure and calculating the minimum free energy, Zuker RNA folding algorithm implement; MFOLD 3.5 software (30) (available at http://mfold.rna.albany.edu/?q=mfold) was used. Target genes for the novel miRNAs were predicted using the web tool psRNATarget (31)(available at http://bioinfo3.noble.org/psRNATarget) and the Plant Ensemble database (available at http://plants.ensembl.org).

### 3.3. Prediction of Potential miRNAs

**Figure 1:**
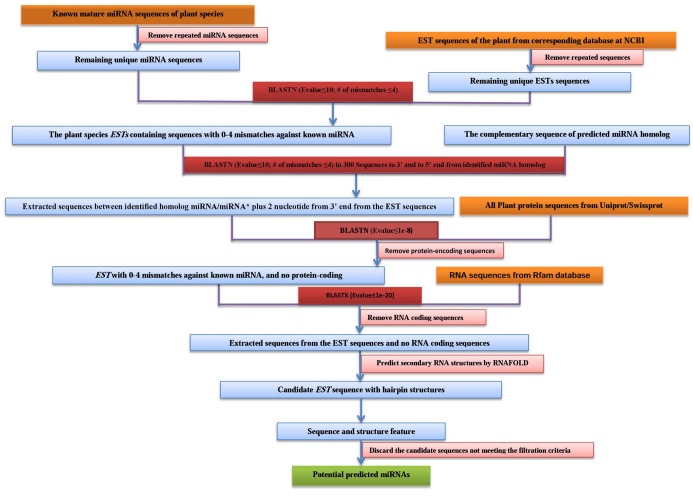
Schematic representation of steps taken to identify homologs of previously reported plant miRNAs.

Potential miRNAs in *B. rapa* were predicted according to (32) with some modification. The workflow for prediction potential conserved miRNAs in *B. rapa* was shown in Figure 1. Redundant sequences were removed from the reference miRNA sequences; remaining unique miRNA sequences from plant species were used as query to search homologous sequences in *B. rapa* EST sequences (6537sequences) via BLASTN (33).

The BLASTN parameters were set as follows: maximum target sequence=1000, expect threshold =10, and the remaining parameters were set as default. All ESTs, with no more than 4 mismatches against the query sequences were saved. The repeated sequences of the same genes were discarded using Codoncode Aligner. The remaining sequences were used to conduct a BLASTX analysis in order to remove the protein-coding sequences. The secondary structures of the selected EST sequences were predicted and generated using Zuker folding algorithm (30) with MFOLD 3.5. The following parameters were used for predicting the secondary structures: folding temperature was fixed at 37°C; ionic condition was set at 1 M NaCl with no divalent ions; maximum interior/ bulge loop size was set as 30; and the grid lines in energy dot plot were turned on, with rest set as default. Retaining sequences were considered as potential miRNA candidates only if they fit the criteria described earlier (32)**.** The secondary structures of predicted pre-miRNAs should have higher MFEI (minimal folding-free energy index) needed for high thermodynamic stability of the sequences to form stable secondary loop structure (34) and negative MFE (minimal free energy) adopted for distinguishing miRNA from other small RNAs (1). MFE of previously identified pre-miRNAs of *B. rapa* ranged from -10.1 to -75.4 kcal.mol^-1^ with an average -39.22 kcal.mol^-1^ (35) therefore predicted pre-miRNAs with MFE lower than -15 kcal.mol^-1^ were discarded. On the other hand, the calculated MFEI for previously identified *B. rapa* precursor miRNAs were -0.349 to -1.210 kcal.mol^-1^ with average of -0.738 kcal.mol^-1^ (35). Accordingly, the predicted precursor miRNAs with up to -0.7 kcal.mol^-1^ were considered. Albeit it is suggested that MFEI values ≤ -0.85 are strongly indicative of actual miRNAs (1,2) (the MFEI was calculated from MFEI = [(MFE /length of the RNA sequence)*100]/(G+C)% based on (22)).The content of G + C were ranged from 20-70% based on average content of previously identified *B. rapa* miRNAs (ranged 18.03-63.27% with average of 40.33% base on (35)). To classify identified potential miRNA genes in to transposon-like and non-transposon-like miRNA gene, RepeatMasker software (available at: http://www.repeatmasker.org/cgi-bin/WEBRepeatMasker) were used. Also to determine the genome location of potential miRNAs, the miRNA precursor sequences were used as queries sequences to BLAST against *B. rapa* genome in plant Ensembl database (http://plants.ensembl.org) with sensitive BLAST parameter settings (cut-off *E*value = 0.01). Also, the corresponding precursor sequences of the candidate *B. rapa* miRNAs were aligned with collected plant pre-miRNA homologs. Phylogenetic trees were constructed using neighbor-joining method. Based on identity percentage evolutionary relationships were illustrated.

### 3.4. Prediction of Potential *B. rapa* mRNA Target Genes

The web tool psRNATarget, an updated version of web-based miRU(http://bioinfo3.noble.org/miRNA/miRU.htm), was applied to predict candidate *B. rapa* miRNA targets. The psRNATarget default criteria were considered.

## 4. Results

### 4.1. Identification of Potential miRNAs

**Figure 2:**
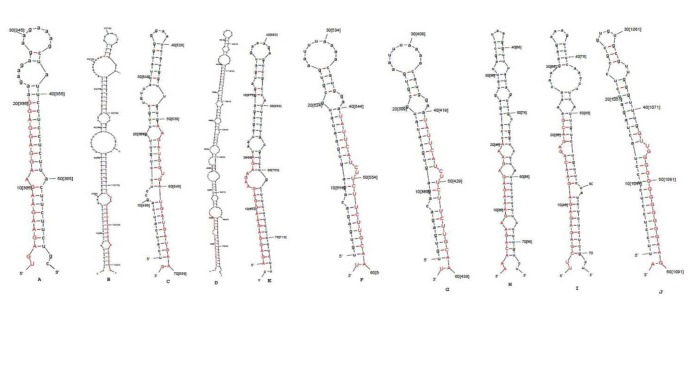
Fold back structure of *B. rapa* representative miRNAs as determined by the RNA Fold program. The mature miRNA sequences are shown by letters in red. A: *miR5021a*, B: *miR5021b*, C: *miR5658*, D: *miR838a*, E: *miR5021c*, F: *miR838b-1*, G: *miR838b-2*, H: *miR156h-5p*, I: *miR2936*, J: *miR2919*.

The miRNA homology-based BLAST searches were made by comparing all unique plant miRNAs with the *B. rapa* EST sequences (3157 sequences) (36). After searching homologous sequences, the redundant sequences of the same genes were removed and the protein coding sequences also were eliminated from the data set. The rest were looked for the formation of hairpin loop secondary structures by MFOLD 3.5 (34) (Fig. 2). Finally, the candidates were manually inspected according to the screening criteria described in the method. Identified pre-miRNA sequences were derived from EST sequences. The pre-miRNA sequences length ranged from 60-178 nt with an average length of 83.8 nt. Similar to other plant species (34), pre-miRNAs of *B. rapa* range from 66-305 nt (35). In total, 10 potential *B. rapa* miRNA sequences belonging to 6 conserved miRNA families were extracted from EST sequences. Novel microRNAs from *B. rapa* were named based on microRNA nomenclature protocol proposed by miRBase (28). Characteristics of the novel miRNAs in* Brassica rapa* are presented in Table S1. The miRNA sequence was subjected as query for BLAST and the loci of the predicted miRNAs on the chromosome were determined (Table 1).

**Table 1:** Genomic location of novel Pre-miRNA sequences predicted from *B. rapa.*

**Bra-MIR**	**Chromosome/Genomic location**	**Alignment score**	**E-value**	**Alignment length**	**Percentage identity**	**Location**
*bra-MIR156h-5p*	Scaffold005103 293 to 365 (+)	365	9.3e-11	73	100	Non protein coding
*bra-MIR156h-5p *	A01 15757343 to 15757415 (+)	347	3.6e-09	73	97.26	Non protein coding
*bra-MIR156h-5p*	A03 2249965 to 2250041 (+)	298	5.9e-07	77	90.91	Exon
*bra-MIR2919*	-	-	-	-	-	-
*bra-MIR2936*	A03 13696574 to 13696641 (+)	310	1.7e-07	71	95.77	Non protein coding
*bra-MIR2936*	A05 9225154 to 9225229 (+)	206	0.0085	76	78.95	Non protein coding
*bra-MIR5021a*	-	-	-	-	-	-
*bra-MIR5021b*	A02 16725864 to 16725971 (+)	522	1.4e-15	108	98.15	Non protein coding
*bra-MIR5021c*	A01 27415853 to 27415928 (+)	380	1.2e-10	76	100	Non protein coding
*bra-MIR5658*	A06 17662015 to 17662085 (+)	319	6.7e-08	71	94.37	Exon
*bra-MIR838a*	A01 4533732 to 4533877 (+)	632	4.9e-22	146	90.41	Intron-exon
*bra-MIR838b-1*	A06 415947 to 416007 (+)	296	7.3e-07	61	98.36	Non protein coding
*bra-MIR838b-2*	A06 415947 to 416007 (+)	296	7.3e-07	61	98.36	Non protein coding

Herein, from 10 identified pre-miRNA sequences belonging to 6 families, 4 miRNAs belonging to 2 miRNAs families were previously reported (miR838 and miR156 families), but the other 6 belonging to 4 families were found as novel miRNAs. However, the novel member of miR838 family had up to 4 mismatch positions with previously reported members of the family in *B. rapa*. Several miRNA families were identified in multiple members (paralogs) within the same plant species, however, it may be due to clone overlap or the same sequence of pre-miRNAs. The chromosomal location survey of the atr-MIR838b-1 and atr-MIR838b-2 revealed that these miRNAs are not regarded as multiple copies of these two identified miRNA genes (Table 1). miRNA members belonging to the same family were not essentially placed on the same arm of the precursor hairpin structure. Accordingly, one of the predicted miRNAs from the miR5021 family was located on 3^’^ arm while the others were located on 5^’^ arm of the corresponding pre-miRNA (Fig. 2). From 10 novel predicted miRNA, 7 mature miRNA sequences had uracil (U) at the start of their 5^’^ tail, which was consistent with previous results in other plants (35,38). It is due to high affinity of Ago to bind to U at the end of mature miRNAs. The G + C content of predicted pre-miRNA sequences ranged from 29.77% to %60 with an average of %42.99. Here, the MFE content of the predicted *B. rapa* miRNA fold back structures were from -15 to -58.6 kcal.mol^-1^ with an average of -27.19 kcal.mol^-1^. Meanwhile, the MFEI values that resulted from structure prediction and calculated for identified pre-miRNA, limited in -0.746 to -1.08 kcal.mol^-1^ with a middle of 0.8211 kcal.mol^-1^.

Loci (9 individual locus) were involved in encoding novel miRNAs were placed in non-protein coding regions, apart from 2 that were located in intragenic region as well as 1 miRNA that were located in intron-exon region of the genome (Table 1). Additionally, *bra-MIR2919* and *bra-MIR5021a* locations in *B. rapa* genome were not identified (Table 1). The matched sequences position in the hairpin resembled to those of their known counterparts.

Transposable elements have the potential to become miRNA genes, particularly inverted repeats that shape hairpin structures (39). Only 2 miRNA genes have repeated elements in their sequences that might be parts of transposable elements (Table 2).

**Table 2:** Repeat content determined in novel *MIRNA* sequences from *B. rapa*.

** Query sequence**	** Begin**	** End **	**C+/match repeat**	** Score **	**Identified in**
bra-miR5021a	3	33	+ (AGA)n	16	EST
bra-miR838a	3 3	3	+ (TTC)n	15	EST
bra-miR838a	112	135	+ (CT)n	18	EST

### 4.2. Phylogenetic Analysis of Conserved Identified miRNAs

**Figure 3:**
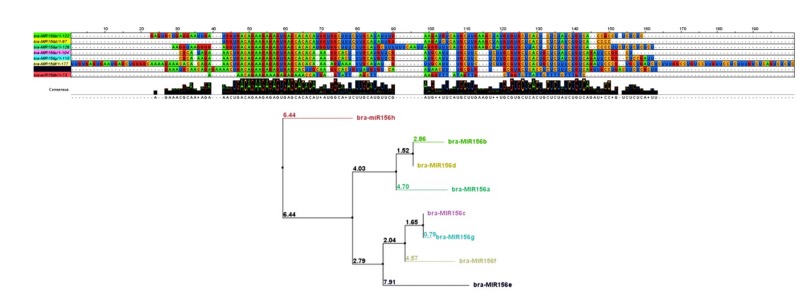
Multiple sequence alignment and phylogenetic tree of the conserved identified bramiR156h- 5p, and its previously known paralogs. The tree was constructed using the neighbour joining method based on the identity percentage model. Numerals at the nods indicate the genetic distance value (%) based on JC69 model.

Predicted mature miRNA sequences were compared with other members in the same family; the comparisons were showed that each of the identified *bra-miR2919*, *bra-miR2936* and *bra-miR5658* had one member identified in *Oryza sativa* (*ora-miR2919*), *Arabidopsis thaliana* (*ath-miR2936*) and *A. thaliana* (*ath-miR5658*) with 4, 2 and 4 mismatch positions, respectively. The conserved identified *bra-miR156h-5p* with several paralogs as reported by (35) had 4 mismatches in comparison with mature sequences of its paralogs. Multiple sequence alignment of the miR156 family precursor in *B. rapa* was performed using ClustalW. Phylogenetic tree was constructed using the neighbor-joining method with default settings, showing the evolutionary relationships (Fig. 3).

The mature miRNA sequence locations were conserved exactly in 14 nucleotide positions once compared between predicted and previously reported miR156 family paralogs. The observed different positions might offer an interconnecting site interacting with protein binding factors. Meanwhile, the two arms of the hairpin were also conserved at the peak of conservation (Fig. 3).

**Figure 4:**
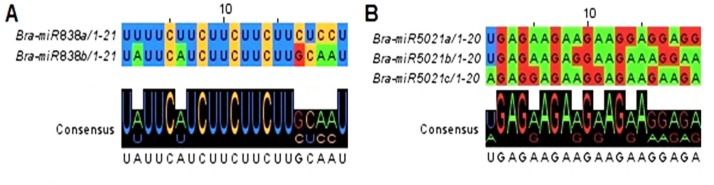
Conservation of identified miR838 (A) and miR5021 (B) families’ mature sequences in *B. rapa*.

Comparison of the miRNA families with previously identified miRNAs presented in miRBase revealed that identified *bra-miR5021* paralogs a, b and c had one ortholog in *A. thaliana* (*ath-miR5021*) with 4 mismatches and some mismatch positions in the mature sequence of miR5021 paralogs (Fig. 4A).

**Figure 5:**
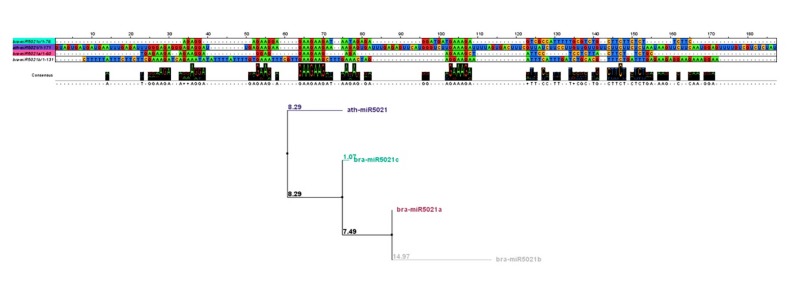
Multiple sequence alignment and phylogenetic tree of the novel *B. rapa* and the member of miR5021 in *Arabidopsis thaliana* miRNA5021 family. The tree was constructed using the neighbour joining method based on the identity percentage model. Numerals at the nods indicate the genetic distance value (%) based on JC69 model.

Multiple sequence alignment of precursor sequences of miR5021 family followed by phylogenetic analysis was indicative of evolutionary relationships of pre-miRNA sequences in *bra-miR5021* miRNAs (Fig. 5).

In this family, despite showing high sequence conservation in mature miRNA sequences, precursor sequences were diverse in fold back structure and sequence length.

**Figure 6:**
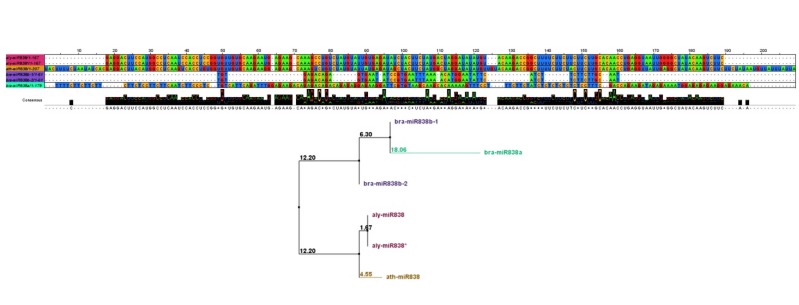
Multiple sequence alignment and phylogenetic tree of the conserved identified 3 members of *B. rapa*, 2 members of *Arabidopsis lyrata* and one member of *A. thaliana* miRNA838 family. The tree was constructed using the neighbour-joining method based on the identity percentage model. Numerals at the nods indicate the genetic distance value (%) based on JC69 model.

*bra-miR838* paralogs a and b had 3 orthologs (*aly-miR838a* and *aly-miR838b* in *Arabidopsis lyrata* and *ath-miR838* in *A. thaliana*) with 4 mismatches (Fig. 4B). The two miRNAs were *bra-miR838b-1* and *bra-miR838b-2.* They were probably clone overlap repeats from different ESTs. Multiple sequence alignment and phylogenetic tree also indicated the evolutionary relationship among pre-miRNA sequences of miR838 family members (Fig. 6).

The diversity in miRNA sequence could also be detected in mature miRNA sequences locations. As mentioned above, the mature sequences in miR838 and miR5021families show some mismatch positions in alignment with identified miRNA sequences in their family. These variations in a few nucleotide positions may give the chance for some miRNAs to base pair with other target mRNAs.

### 4.3. Identification of *B. rapa* mRNA Target Genes

According to the perfect or near perfect sequence complementarity amongst miRNAs and their targets, psRNATarget tool was employed for prediction of *B. rapa* target genes. By applying the predicted mature miRNA sequences in *B. rapa* as query in psRNATarget, and by searching the obtained targets in *B. rapa* genome in Ensemble plant (http://plants.ensembl.org), several protein coding targets belonging to several gene families as well as several targets with unknown functions were identified for these miRNAs. The results showed that each of identified miRNAs regulates more than one protein coding gene usually by cleavage of mRNA and rarely by suppression of translation (Table S1). Interestingly, no target genes were detected for the novel *bra-miR2919*. It may be the result of incomplete *B. rapa* sequence database or it is target-less miRNA that is evolutionarily transient. All of identified miRNAs (except for miR2919) target transcription factors, which directly or indirectly have effect on transcription process with varieties of regulatory functions in different pathways (Table S2). Diverse protein coding genes having duty in translation, stress response, structural component, development, and metabolism were predicted as target genes for predicted miRNAs in *B. rapa*. For instance, myeloblastosis (MYB) families of transcription factors were identified as *bra-miR5021a* and *bra-miR838a* target genes. MYBs have key roles in regulating plant development, metabolism, hormone response during seed development and germination, signal transduction, cellular proliferation and differentiation, and response to environmental abiotic and biotic stresses (for most details see (40). In addition, various enzymes were targeted by these miRNAs indicating critical role of these miRNAs in metabolism. Enzymes such as acetyl-CoA C-acyltransferase (GO: 0003988), phosphoprotein phosphatase (GO: 0004721) control the state of protein’s phosphorylation and regulate cellular activities, and threonine kinase were targeted by *bra-miR5021a*. *bra-miR5021b* is involved in regulation of kinases (GO: 0004672) such as S-locus protein kinase, transmembrane protein kinase, and catalytic/ hydrolase. Aspartyl protease transcripts were the target of *bra-miR5658*. *bra-miR838a* regulates the translation of peptidyl-prolyl cis-trans isomerase (GO:0003755), pseudouridine synthase (GO: 0001522), and helicase (GO:0004386) genes. Additionally, cysteine protease inhibitor, pseudouridine synthase and ATPase family protein were targeted by *bra-miR838a*. Moreover, cysteine protease inhibitor, serine-type endopeptidase (GO:0008236), cyclin-dependent protein kinase inhibitor (GO:0007472), laccase; aspartyl protease family; α-(1→4)-glycosyltransferase family protein, polygalacturonase, and polygalacturonate 4-alpha-galacturonosyltransferase (GO:0047262) were regulated by *bra-miR5021c*. It was also found that endonuclease/exonuclease and photolyase transcriptome were targeted by *bra-miR838b* as well as threonine kinase, S-acyltransferase (GO:0016417) that catalyze the transfer of an acyl group to a sulfur atom on the acceptor molecule, histidine phosphotransfer kinase (GO:0009927), which act as a phospho-His intermediate (involved in transfer a phosphate group between a kinase and a response regulator), and hydrolase (GO:0016787) with role in hydrolysis of various bonds, *e.g.* carbon–oxygen, carbon–nitrogen, carbon–carbon, phosphoric anhydride bonds, regulated by *bra-miR156h-5p*. The results demonstrated that a mature miRNA sequence may regulate hundreds of different protein coding transcripts as well as a given transcribed gene might be regulated by multiple mature miRNAs. Our results also support the previous suggestion that demonstrate miRNA regulatory effects should be focused on regulatory networks rather than individual connections between miRNA and their target genes.

### 5. Discussion

In eukaryotic cells, not all transcribts are protein-coding RNAs. These molecules are not limited to transfer RNA (tRNA), ribosomal RNAs (rRNA), small nucleolar RNAs (snRNAs) and small nucleolus RNAs (snoRNA). MicroRNAs form another class of such non-protein coding molecules. Functionally and despite the fact that many of which do not have any known clear cut cellular roles (42), some others such as miRNA genes do have regulatory function; controlling overall transcription status, bring gene networks into homeostasis. Generally speaking, mature miRNAs are looking conserved in terms of both sequence and structure in plants (2). This means, the chance of finding orthologous sequences across plant genera are quite high that justifies homology searches according to ESTs for prediction of unidentified miRNA sequences (19,36), an approach that is taken into consideration in bean (26), cassava (25), potato (24), maize (21), tomato (22), plant switch grass (23), *Amborella* (32), Chlamydomonas (41) and many others. Most of miRNAs predicted by this method can be obtained by high-throughput deep sequencing process (37), something that have so far been shown that many of the predicted such molecules are genuine pre-miRNAs (32) that are able to transcript in different developmental and/or time position. Likewise, it is demonstrated that most of miRNAs predicted by *in silico* methods are in accordance with the experimentally predicted miRNAs (43). Here, putative miRNAs for *B. rapa* were identified accordingly; eight of which were resided in intergenic and two were in intragenic regions, known as mitrons. Mirtrons are not common in plants (25) and their biogenesis is different from other miRNAs, requiring transcription of relevant gene residing and splicing the intron bearing the mitron, similar to the protein-coding transcripts (44). Their precursor sequence length is shorter than with canonical pre-miRNA. Mirtron precursors are spliced during mRNA processing instead of Dicer-mediated cleavage (44). In addition to predicting plant mitrons, 2 EST-derived miRNAs with low complexity and repetitive sequence region were detected. Something that can be related back to the miRNAs derived from transposable elements and sequence repeats (45).

The miRNA target(s) recognition allows predicting the miRNAs regulatory role in cellular function and gene regulation network. In plants, miRNAs control the expression of the genes encoding transcription factors, stress response proteins and other proteins that impact development, growth and physiology of plant either by cleavage of mRNA or suppression of translation of the targets. Our data were indicative that in *B*. *rapa* most of putative miRNAs regulates more than one protein coding gene mostly by cleavage of mRNA. Interestingly, no target genes were detected for the novel *bra-miR2919*. It may be the result of incomplete *B. rapa* sequence database or it is target-less miRNA that is evolutionarily transient. All others directly or indirectly had effect on transcription process with varieties of regulatory functions in different pathways. Most of the target sequences were parts of the gene regulatory networks.

## 5. Conclusion

MicroRNAs have diverse regulatory functions that occur at post-transcriptional stage. Comprehension of miRNA impact can unravel some of the unanswered mysteries of biology, shedding light on the overall gene regulatory network. Recently, research in miRNA led to the prediction of many miRNA genes and their regulating transcripts from diverse species (32,41). However, many unknown miRNAs stay to be discovered and functionally annotated. Due to high conservation in plant miRNAs, novel homologs miRNAs can be eidentified in other plant species through sequence and structural homology. Furthermore, search for the complementary sequences within genome allows prediction of miRNA target site(s). Here, we constructed EST-based homology search in *B. rapa* (Field mustard) for identification of potential miRNAs by considering a series of filtration criteria, and highlighted potential identified miRNAs targets. This analysis led to the identification of 10 different conserved sequences that fall into 6 miRNA families. The identification of the novel evolutionary conserved micro RNA genes and their regulatory transcripts in *B. rapa* is anticipated to provide baseline information for further search about the biological functions and evolution of plant miRNAs.
